# Clinical and cVEMP Evaluation Predict Short-Term Residual Dizziness After Successful Repositioning in Benign Paroxysmal Positional Vertigo

**DOI:** 10.3389/fmed.2022.881307

**Published:** 2022-05-24

**Authors:** Chun-Yan Jiang, Jing Wu, Liang Shu, Xu-Hong Sun, Hui Pan, Qian Xu, Si-Cheng Wu, Jian-Ren Liu, Yun Li, Wei Chen

**Affiliations:** ^1^Department of Neurology, Shanghai Ninth People's Hospital, Shanghai Jiao Tong University School of Medicine, Shanghai, China; ^2^Department of Neurology, Huangpu Branch, Shanghai Ninth People's Hospital, Shanghai Jiao Tong University School of Medicine, Shanghai, China; ^3^Biostatistics Office of Clinical Research Center, Shanghai Ninth People's Hospital, Shanghai Jiao Tong University School of Medicine, Shanghai, China; ^4^Department of Otolaryngology-Head and Neck Surgery, Shanghai Ninth People's Hospital, Shanghai Jiao Tong University School of Medicine, Shanghai, China; ^5^Hearing and Speech Center, Shanghai Ninth People's Hospital, Shanghai Jiao Tong University School of Medicine, Shanghai, China

**Keywords:** benign paroxysmal positional vertigo, residual dizziness, dizziness handicap inventory, cervical vestibular evoked myogenic potential, visual analog scale

## Abstract

**Objective:**

Residual dizziness (RD) is a frequent symptom with unknown pathogenesis, often complained about by the patients with benign paroxysmal positional vertigo (BPPV), even after a successful canalith repositioning procedure (CRP). This study aims to quantitatively evaluate the short-term RD severity and its risk factors in patients with BPPV after successful CRPs.

**Methods:**

In total two hundred and twenty patients with BPPV after successful CRPs (W0) were prospectively followed up for 1 week (W1). Besides demographics and serial neuropsychological assessments (including dizziness handicap inventory-DHI, etc.), patients also received cervical/ocular vestibular evoked myogenic potential (c/oVEMP) evaluation. RD was defined as patients with dizziness or imbalance, dizziness visual analog scale (VAS) >1, and without positional vertigo or nystagmus at W1. Demographic, clinical, and VEMPs differences were compared among the three groups: patients with minor (dizziness VAS 1–3) and moderate-to-severe RD (dizziness VAS > 3) and without RD.

**Results:**

The total frequency of RD at W1 was 49.1% (*n* = 108), with 32.3% (*n* = 71) minor, and 16.8% (*n* = 37) moderate-to-severe RD. Logistic regression analyses revealed that RD was closely associated with DHI status (OR = 2.101, *P* = 0.008) at W0, this effect was not present for minor RD. In addition to DHI score > 30 (OR = 4.898, *P* < 0.001) at W0, bilateral cVEMP absence (OR = 4.099, *P* = 0.005) was also an independent influential factor of moderate-to-severe RD.

**Conclusion:**

Our study highlights the importance of RD quantified evaluation. DHI score >30 and bilateral cVEMP absence could increase the risk of short-term moderate-to-severe RD.

## Introduction

As one of the most common peripheral vestibular diseases, benign paroxysmal positional vertigo (BPPV) is caused by detached otoconia falling from the utricle to semicircular canals (1). Although most of the patients with BPPV can be treated through an appropriate canalith repositioning procedure (CRP) (1, 2), some patients still experience a sense of dizziness or imbalance without positional vertigo or nystagmus, which is also known as residual dizziness (RD). It usually occurs within the first month after CRP and lasts for days to weeks (3–6). The existence of RD has impacted the patients' work and quality of life (5, 6). The reported prevalence of RD ranged from 31 to 61% (7), depending upon the time of evaluation, enrolled subjects, and the definition of RD. At present, there are no universal diagnostic criteria for RD as it is a relatively subjective symptom, and objective quantified evaluation is lacking.

The pathogenesis of RD is still unknown. Several factors have been reported to be associated with RD, including elderly onset age (8), the duration of vertigo before CRP (3), psychological comorbidities (5, 8), autonomic dysfunction (6), and otolithic organ disorders (9–12). Among these factors, one of the most relevant factors is otolithic organ dysfunction, which could be reflected by cervical/ocular vestibular evoked myogenic potentials (c/oVEMPs). However, the results from VEMP research are inconsistent. Yetister in 2014 found that a decreased cVEMP response in the affected ear may be associated with longer symptom persistence after CRP (9); Seo et al. in 2017 reported that a persistent reduced oVEMP response in the affected side is related to RD occurrence (11). Whereas researchers from Korea in the year 2019 found that augmented response of cVEMP in the affected side may predict the development of RD (12).

Therefore, we conducted a prospective 1-week follow-up study among BPPV patients after successful CRPs to explore the occurrence of RD, RD severity, and related risk factors so as to provide evidence for personalized treatment.

## Materials and Methods

### Patients

**Figure 1:**
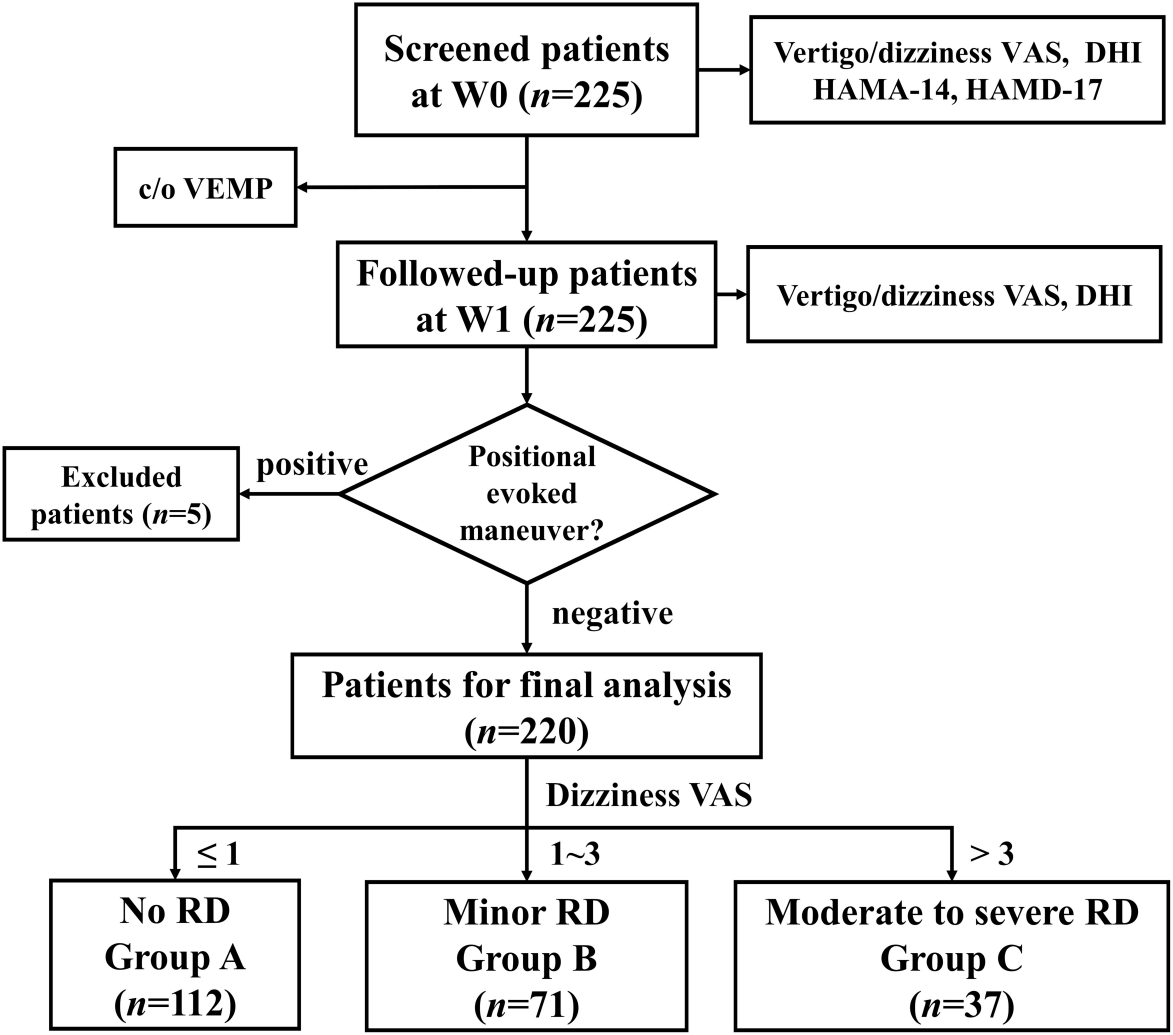
The flowchart of the study. VAS, visual analog scale; DHI, dizziness handicap inventory; HAMA-14, Hamilton Anxiety Scale-14; HAMD-17, Hamilton Depression Scale-17; c/o VEMP, cervical/ocular vestibular evoked myogenic potential; W0, at enrollment; W1, 1 week after enrollment; RD, residual dizziness.

Patients with BPPV came from our neurology vertigo outpatient clinic from June 2018 to October 2019. The diagnosis of idiopathic BPPV conformed to Barany criteria (13) and the American Academy of Otolaryngology-Head and Neck Surgery Guideline (14). In summary, it requires: (i) recurrent, transient dizziness or vertigo occurring after the change of head position, which usually lasts no more than 1 min, (ii) episodic vertigo and characteristic positional nystagmus occurring in the positional-evoked maneuvers: Vertical jumping nystagmus with torsion component elicited after a latency of one or few seconds by the Dix-Hallpike maneuver, which typically lasts <1 min, can be determined as canalolithiasis of the posterior canal (pc-BPPV); horizontal geotropic direction-changing nystagmus elicited after a brief latency or no latency by the roll test, which typically lasts <1 min, can be determined as canalolithiasis of the horizontal canal (hc-BPPV). Occasionally, horizontal apogeotropic nystagmus lasting <1 min could also be canalolithiasis of hc-BPPV. The horizontal apogeotropic direction-changing nystagmus elicited after a brief latency or no latency by the roll test, which lasts more than 1 min, can be determined as cupulolithiasis of the horizontal canal (hc-BPPV-cu). Each patient with BPPV underwent corresponding CRPs immediately after the diagnosis. Patients with posterior canal BPPV were treated with Epley's maneuver, while those with horizontal canal BPPV were treated with Barbecue's or Gufoni's maneuvers. Inclusion criteria of this study were as follows: (i) unilaterally posterior or horizontal canal BPPV; (ii) the onset age ranged from 18 to 80 years old; (iii) positional vertigo and nystagmus disappeared after 1–3 times of CRPs at the first visit (W0), indicating successful repositioning treatment; (iv) patients completed face-to-face interview 1 week(W1) after CRPs, as this time point was regarded as short-term evaluation, according to the Chinese and American BPPV diagnostic and treatment guidelines (14, 15). Positional-evoked maneuvers were repeated at W1. The following patients were excluded: (i) patients had central positional vertigo/nystagmus, which did not beat in the plane of the affected canal; (ii) BPPV recurred at W1 with the positive Dix–Hallpike test or the Roll test; (iii) previous history of deafness, the Meniere's disease, vestibular neuritis, vestibular migraine, severe brain trauma, cerebrovascular disease, epilepsy, drug dependence, schizophrenia, and other serious mental diseases, and organ dysfunction. We screened 225 patients with BPPV at W0, 5 patients were excluded because of the positive positional-evoked maneuver at W1. Finally, 220 patients were enrolled for analyses (Figure 1).

This study was approved by the Ethics Committee of Shanghai Ninth People's Hospital, Shanghai Jiao Tong University School of Medicine (2018-128-T106). All the enrolled patients signed the informed consent forms. All the methods were carried out in accordance with the relevant guidelines and regulations.

### Clinical Profiles

We collected patient demographic data during the first visit: age, gender, duration of vertigo before treatment, affected canal, and frequency of CRPs at W0. A visual analog scale (VAS) was used to assess the severity of dizziness and vertigo separately. Vertigo VAS and dizziness VAS are commonly used scales in BPPV clinical practice (16). It ranged from 0 (absence of symptoms) to 10 (very severe symptoms). Dizziness handicap inventory (DHI) was employed to quantify the impact of dizziness on the patient's quality of life. It consists of 25 items, each with the option of “yes,” “sometimes,” and “none,” 4, 2, or 0 points were assigned, respectively. It covers 3 subdomains: functional (DHI-F), emotional (DHI-E), and physical (DHI-P) scores. DHI's total score ranges from 0 to 100 points. The higher the score, the greater the impact on the patient's daily life (17). According to the report from Whitney SL (18), DHI > 30 indicates moderate-to-severe abnormality (18). In addition, we also evaluated psychological conditions with Hamilton Anxiety Scale-14(HAMA-14) (19) and Hamilton Depression Scale-17(HAMD-17) (20). HAMA-14 score ≥8 indicates anxiety symptoms, and the HAMD-17 score ≥8 indicates depressive symptoms. For the aforementioned scales, the DHI and VAS scales were evaluated both at W0 and W1. Whereas, HAMA-14 and HAMD-17 were evaluated only at W0 (Figure 1).

RD is a relatively subjective symptom. Previous studies lacked a quantitative assessment of RD. In our study, RD was defined at W1 as follows: 1) patients still had dizziness or imbalance without positional vertigo or nystagmus, as determined by positional-evoked maneuvers; 2) VAS score for dizziness was greater than 1 point (21). Patients without RD were defined as Group A. As principles in pain research (22), RD was furtherly stratified into minor (dizziness VAS 1–3, Group B) and moderate-to-severe (dizziness VAS>3, Group C) RD.

### VEMPs Evaluation

VEMPs were evaluated for each participant within 1 week after enrollment. All the VEMP evaluation was performed in our hearing center, Department of Otolaryngology-Head and Neck Surgery, Shanghai Ninth People's Hospital, Shanghai Jiao Tong University School of Medicine. It is the Chinese national quality control center of neuro-otological examinations. We have specialized technicians to undertake VEMP tests, which relatively guarantees the quality of VEMP. The VEMP recordings were done using the auditory-evoked potential analyzer (Neuro-Audio, Neurosoft LLC, Ivanov, Russia). The stimuli cosisted of a 500 Hz tone burst. An alternating tone-burst polarity was administered with a repetition rate of 5.1/s, and 60–200 sweeps were averaged after rejecting the artifacts using IP-30 insert phones. The high and low pass filters were set at 8 and 1,500 Hz. Only electrode impedances less than 5 kOhm were accepted. The recordings were carried out in a sound-treated room.

In the cVEMPs test, all subjects were placed in a supine position and asked to rotate their head away from the stimulated side to record electromyographic activity over the activated sternocleidomastoid muscle (SCM). Surface EMG activity was recorded with superficial electrodes placed on the middle third of the SCM, with the reference electrode placed on the upper third of the sternum and the ground electrode on the middle of the forehead. In the oVEMP test, all the subjects were lying in one position and were instructed to look upper medially at a small fixed target 1 m from the eyes. The visual angle was ~30°, which has been found to elicit the largest responses compared with other eye positions. In our lab, the electrode was placed on a rectangular paster. The active electrodes were placed on the face, oriented vertically and ~1 cm below the center of the lower eyelid just inferior to the contralateral eye for sound stimulation. The superior margin of the reference electrode paster was placed about 1 cm below the inferior margin of the active electrode paster on the cheek, and the ground electrode was placed on the forehead. Each subject's eyes remained fixed on the target throughout the test.

An absent response was noted as the main outcome when a typical bidirectional waveform was not observed at the maximum stimulus intensity (110dB nHL) in our center. We also measured the detailed parameters, such as threshold, latency, amplitude, and interaural amplitude difference (IAD) ratio of c/oVEMP, in patients with bilaterally elicited waves. The IAD ratio was defined as follows: (unaffected side ear amplitude - affected side ear amplitude) ÷ (affected side ear amplitude + unaffected side ear amplitude).

### Statistical Analysis

SPSS 22.0 (version 23.0 for Windows) was used for statistical analysis. GraphPad Prism (version 5.0 for Windows) was used for plotting the graphics. We used mean and SD for continuous variables (age, etc.) with a normal distribution, median and interquartile range (IQR, 25th−75th percentile) for those with skewed distributions. Percentages in the row of each categorical variable were exhibited in the table. To compare categorical data among the three groups, we applied the chi-square test or Fisher's exact test. Independently associated factors for RD were analyzed by three binary logistic regressions. *P* < 0.05 was defined as statistically significant.

## Results

### Demographic Data and RD Incidence

The age of the enrolled patients ranged from 25 to 80 years, with an average age of 57.2 years. There were 55 men and 165 women, and the ratio of men to women was 1:3. There were 163, 51, and 6 cases of pc-BPPV, hc-BPPV, and hc-BPPV-cu, respectively. A total of 100 and 79 patients had it for the first time, whereas 41 subjects had a history of BPPV. The median duration of vertigo before CRP is 7 (4–14) days. At W1 evaluation, 108 subjects developed RD, and the incidence of RD was 49.1%, with 32.3% (*n* = 71) minor, and 16.8% (*n* = 37) moderate-to-severe RD.

### Clinical Correlates With RD and Its Subtypes

**Figure 2:**
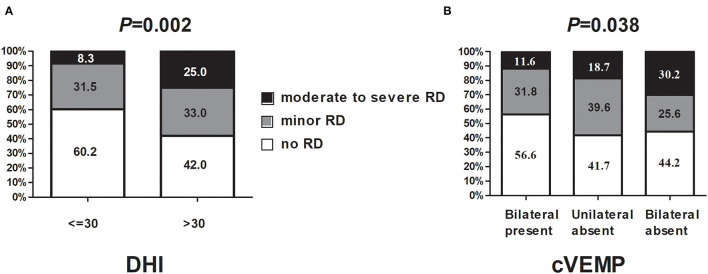
The relationship between DHI, cVEMP absence, and RD severity. **(A)** DHI correlates with RD and its subtypes; **(B)** cVEMP correlates with RD and its subtypes. DHI, dizziness handicap inventory; cVEMP, cervical vestibular evoked myogenic potential.

As shown in Table 1, there was an obvious difference in DHI total score among the three groups (*p* < 0.001) at W0. The DHI-P and DHI-F score was significantly increased in Group C. After CRPs, the total DHI score decreased significantly. The total trend of DHI and dizziness VAS is consistent. Patients with a DHI score > 30 at W0 had a higher frequency of moderate-to-severe RD at W1, relative to those with a DHI score ≤ 30 (25.0 vs. 8.3%, *p* = 0.002, Figure 2A). There was no significant difference in age, gender distribution, vertigo duration, the number of CRPs, involved semicircular canal, anxiety, and depressive score among the three groups. Binary logistic regression analysis revealed that RD was closely associated with DHI >30 (OR = 2.101, *p* = 0.008) at W0, this effect was not present for minor RD. Whereas, for moderate-to-severe RD, the odds ratio of DHI > 30 increased to 4.898 (Table 3).

**Table 1:** Clinical characteristics among BPPV patients with minor, moderate-to-severe, and without RD.

**Category**	**No RD Group A**	**Minor RD** **Group B**	**Moderate- to-severe RD Group C**	***p* value**
*n*	112	71	37	
**Age**, *n* (%)				0.556
>65 year	29 (46.8)	20 (32.3)	13 (21.0)	
≤ 65 year	83 (52.5)	51 (32.3)	24 (15.2)	
**Gender**, *n* (%)				0.400
Female	82 (49.7)	52 (31.5)	31 (18.8)	
Male	30 (54.5)	19 (34.5)	6 (10.9)	
**Vertigo duration**, *n* (%)				0.485
>7 days	51 (51.0)	35 (35.0)	14 (14.0)	
≤ 7 days	59 (50.4)	35 (29.9)	23 (19.7)	
**CRPs**, *n* (%)				0.805
Multiple	38 (49.4)	24 (31.2)	15 (19.5)	
Single	72 (52.6)	43 (31.4)	22 (16.1)	
**Involved canal**, *n* (%)				0.952
Posterior	84 (51.5)	52 (31.9)	27 (16.6)	
Horizontal	28 (49.1)	19 (33.3)	10 (17.6)	
**W0**				
DHI total score	28 (16–44)	34 (20–44)	44 (32–55)	<0.001***
DHI-P	10 (6–16)	10 (7–16)	16 (8–20)	0.015*
DHI-F	14 (6–24)	14 (8–22)	22 (16–26)	0.001**
DHI-E	4 (0–8)	4 (2–12)	4 (2–11)	0.055
DHI total score > 30, *n* (%)	47 (42.0)	37 (33.0)	28 (25.0)	0.002**
DHI total score ≤ 30, *n* (%)	65 (60.2)	34 (31.5)	9 (8.3)	
HAMA-14 score ≥ 8, *n* (%)	19 (40.4)	16 (34.1)	12 (25.5)	0.117
HAMA-14 score <8, *n* (%)	93 (53.8)	55 (31.8)	25 (14.4)	
HAMD-17 score ≥ 8, *n* (%)	16 (45.7)	12 (34.3)	7 (20.0)	0.753
HAMD-17 score <8, *n* (%)	96 (51.9)	59 (31.9)	30 (16.2)	
Vertigo VAS	9 (7–10)	9 (7–10)	10 (8–10)	0.073
Dizziness VAS	2 (0–5)	3 (1–5)	5 (3–7)	<0.001***
**W1**				
DHI total score	1 (0–6)	10 (4–18)	26 (18–40)	<0.001***
Vertigo VAS	0	0	0	NA
Dizziness VAS	0	2 (2–3)	5 (5–5)	<0.001***

BPPV, benign paroxysmal positional vertigo; RD, residual dizziness; DHI, dizziness handicap inventory; DHI-P, DHI-physical subdomain; DHI-F, DHI-functional subdomain; DHI-E, DHI-emotional subdomain; W0, at enrollment; W1, one week after enrollment; HAMA-14, Hamilton Anxiety Scale-14; HAMD-17, Hamilton Depression Scale-17; VAS, visual analog scale; NA, not applicable; CRP, canalith repositioning procedure; *p < 0.05; **p < 0.01; ***p < 0.001. Bold values means P value was statistically significant.

### VEMP Correlates With RD and Its Subtypes

For cVEMP, there were 129 (58.6%), 48 (21.8%), and 43 (19.5%) subjects with bilateral presence, unilateral absence, and bilateral absence, respectively. Concerning oVEMP, there were 87 (39.5%), 47 (21.4%), and 86 (39.1%) patients with bilateral presence, unilateral absence, and bilateral absence, separately. The absence rate of oVEMP was significantly higher than that of cVEMP, both on the ipsilateral (50.0 vs. 30.7%, *p* < 0.001) and contralateral (49.5 vs. 30.3%, *p* < 0.001) side.

Regarding cVEMP, the absence rate was associated with RD whatever on the ipsilateral, contralateral, either, or bilateral sides (Table 2). Patients with cVEMP absence on the bilateral side also had a higher frequency of moderate-to-severe RD at W1, relative to those with bilateral cVEMP presence (30.2 vs. 11.6%, *p* = 0.038, Figure 2B, Table 2). With respect to the absence rate of oVEMP, there was no significant difference among the three groups (Table 2). Also, the threshold, latency, amplitude, and IAD ratio of c/oVEMP were not associated with RD in patients with the bilateral elicited response (Supplementary Tables 1, 2). Taken together, it seems that the main influence on RD came from cVEMP, and not from oVEMP.

**Table 2:** VEMP absence rate among BPPV patients with minor, moderate-to-severe, and without RD.

**Category**	**No RD Group A**	**Minor RD** **Group B**	**Moderate- to-severe RD Group C**	***p* value**
*n*	112	71	37	
**cVEMP**, *n* (%)				
Ipsilateral absence	31 (44.9)	20 (29.0)	18 (26.1)	0.046*
Ipsilateral presence	81 (53.6)	51 (33.8)	19 (12.6)	
Contralateral absence	28 (41.8)	21 (31.3)	18 (26.9)	0.026*
Contralateral presence	84 (54.9)	50 (32.7)	19 (12.4)	
Unilateral or bilateral absence	39 (42.9)	30 (33.0)	22 (24.2)	0.030*
Bilateral presence	73 (56.6)	41 (31.8)	15 (11.6)	
Bilateral absence	19 (44.2)	11 (25.6)	13 (30.2)	0.038*
Unilateral absence	20 (41.7)	19 (39.6)	9 (18.7)	
Bilateral presence	73 (56.6)	41 (31.8)	15 (11.6)	
**oVEMP**, *n* (%)				
Ipsilateral absence	50 (45.5)	38 (34.5)	22 (20.0)	0.227
Ipsilateral presence	62 (56.4)	33 (30.0)	15 (13.6)	
Contralateral absence	51 (46.8)	39 (35.8)	19 (17.4)	0.451
Contralateral presence	61 (55.0)	32 (28.8)	18 (16.2)	
Unilateral or bilateral absence	61 (45.9)	47 (35.3)	25 (18.8)	0.179
Bilateral presence	51 (58.6)	24 (27.6)	12 (13.8)	
Bilateral absence	40 (46.5)	30 (34.9)	16 (18.6)	0.480
Unilateral absence	21 (44.7)	17 (36.2)	9 (19.1)	
Bilateral presence	51 (58.6)	24 (27.6)	12 (13.8)	

RD, residual dizziness; BPPV, benign paroxysmal positional vertigo; cVEMP, cervical vestibular evoked myogenic potential; oVEMP, ocular vestibular evoked myogenic potential; *p < 0.05. Bold values means P value was statistically significant.

To further explore the independent influential factor of RD, three binary logistic regression models were conducted. There was collinearity between the ipsilateral and contralateral absence of c/o VEMP. It revealed that, compared with subjects with bilateral presence, cVEMP absence on either side (OR = 3.136, *p* = 0.006) was an independent influential factor of moderate-to-severe RD at W1, in addition to DHI >30 (Supplementary Table 3). When cVEMP was dichotomized into three subgroups, bilateral cVEMP absence (OR = 4.099, *p* = 0.005) was the real independent associated factor of moderate-to-severe RD at W1 (Table 3), but not unilateral cVEMP absence (*p* = 0.103), relative to subjects with bilateral presence.

**Table 3:** Logistic regression analyses for RD and its severity in patients with BPPV when cVEMP was dichotomized into three subgroups.

**Variables**	**β**	**OR**	**95%CI**	***p* value**
**Model 1: dependent factor: Group (B+C) vs. Group A**				
DHI score > 30	0.742	2.101	1.218–3.623	0.008**
**Model 2: dependent factor: Group B vs. Group A**				
None				
**Model 3: dependent factor: Group C vs. Group A**				
DHI score> 30	1.589	4.898	2.027–11.837	<0.001***
cVEMP			
Bilateral presence		1.0 (reference)	
Unilateral absence Bilateral absence	0.848 1.411	2.334 4.099	0.843–6.461 1.548–10.857	0.103 0.005**

RD, residual dizziness; BPPV, benign paroxysmal positional vertigo; DHI, dizziness handicap inventory; cVEMP, cervical vestibular evoked myogenic potential; β represents regression coefficient; OR, odds ratio; CI, confidential interval; **p < 0.01; ***p < 0.001. Bold values means P value was statistically significant.

There was a correlation between DHI total score at W0 and HAMA-14(*r* = 0.459, *p* < 0.001) and HAMD-17 score (*r* = 0.483, *p* < 0.001); however, DHI total score at W0 was not associated with cVEMP absence(*p* = 0.963) or the duration from onset to treatment of BPPV (*p* = 0.376).

## Discussion

This is the first study worldwide to quantify and stratify the evaluation of RD with sufficient sample size. Based on this nested case-control study with the detailed clinical and VEMP evaluation, we found that DHI >30 is a practical clinical index to predict short-term RD in BPPV; in addition, bilateral cVEMP absence is also an important contributor to moderate-to-severe RD.

DHI has been widely used in the evaluation of the vertigo-related quality of life since it was developed by Jacobson and Newman in 1990. Our group also validated the reliability of DHI sub-items in screening BPPV (23). For RD, Martellucci et al. (24) found that a high DHI score, especially the emotional sub-score at baseline was correlated with a high RD incidence after CRP of BPPV. This study is the second one conducted worldwide to investigate the effect of the initial DHI score on RD. We found that patients with BPPV a DHI score >30 were more likely to develop RD, especially moderate-to-severe RD, indicating that DHI > 30 may be a simple and practical tool for predicting the occurrence of RD. DHI score did not correlate with VEMP abnormality in our study and Strupp's report from Germany in 2018 (25), it may be related to central adaptation. It was reported that BPPV with delayed diagnosis could induce central adaptation. Despite a successful CRP, the inability of quick re-adaptation of the brain after resolution results in persistent RD (26). We propose that patients with a higher DHI score could potentially have more established central adaptation, contributing to RD. Further neuroimaging studies are warranted in the future to investigate the relationship between initial DHI score, central vestibular structural, and functional abnormalities in patients with BPPV.

VEMPs have been widely used for the evaluation of otolith organs and related vestibular pathways since the report from Halmagyi and Colebatch (27). cVEMP and oVEMP could reflect saccular and utricular functions, respectively. It is reported that decreased VEMPs response, especially oVEMP abnormality, plays an important role in BPPV occurrence and recurrence. Whereas, only a few studies are focused on VEMP evaluation and RD (9, 11, 12, 28). The lack of stratified design and limited sample size may explain the inconsistency around whether a decreased or an augmented cVEMP response is truly related to RD as noted in the previous literature. The results of our study showed that as long as the oVEMP or cVEMP was elicited, there was no significant difference in the detailed parameters (such as threshold, latency, amplitude, and IAD ratio) on the affected side or the unaffected side among the three groups (Supplementary Tables 1, 2). These results indicate that the abnormalities of VEMPs in patients with BPPV presented as a type of “all or nothing” characteristic, that is, either the waveform of VEMPs could not be elicited, or the waveform of VEMP was normal, which was consistent with many previous studies (29–31). We also found that there was no significant correlation between the absent side of VEMPs and the affected side of the ear, which is in accordance with the report from Wu et al. (28). Although most patients with BPPV are unilaterally implicated, their otolith dysfunction is often bilateral (10, 32, 33). Our study based on the 220 patients found that bilateral absent cVEMP response was related to moderate-to-severe RD, indicating that the cVEMP pathway is more important than the oVEMP pathway for moderate-to-severe RD. The potential mechanism is still unknown as the vestibulospinal tract is an important component of the cVEMP pathway, not present in the oVEMP pathway (34). It is involved in the body's postural reflexes to maintain balance (35). Patients with absent cVEMP response may be more prone to imbalance even after successful CRPs in patients with BPPV, as imbalance is an important clinical characteristic of RD. Therefore, we propose that bilateral cVEMP absence may be a reliable electrophysiological marker for RD occurrence.

Our results have important clinical implications for personalized BPPV treatment. We do not need further treatment for minor RD, as the clinical characteristics were consistent between BPPV with minor and without RD. Furthermore, we need to identify the patients with DHI >30 or bilateral cVEMP absence at the initial diagnosis, since these patients are prone to develop short-term moderate-to-severe RD. In addition to CRP, vestibular rehabilitation or adjuvant drugs treatment may be required for enhancing the recovery of this subtype of patients. Although the sample size is quite big, we admit that there are some weak points. For VEMP evaluation, patients with BPPV only received one time of evaluation after CRPs, and we did not have the VEMP data from the healthy controls and the patients before CRPs. Therefore, we did not know whether VEMP loss was a temporary or persistent finding of BPPV; the Caloric test, video-head impulse test, and hearing test were not performed for each patient. Whether semicanal paresis was related to RD merits further investigation. In this study, RD was defined by subjective scales, more objective equipment, such as computerized dynamic posturography, to evaluate imbalance is needed in future research (8).

Previous studies have shown that the duration of vertigo before treatment could be a risk factor for RD (5). However, there was no such association in our study. This may be related to the relatively short duration before treatment. The median duration of vertigo before treatment was 7 days in our study, which was shorter than the 10.9 days reported in the literature (24). Although previous studies found that RD may be associated with anxiety and depressive symptoms, HAMD-17 and HAMA-14 scores at the first diagnosis in our study were associated with DHI total score, however, they were not statistically significant among the three groups. Whether psychological factors and otolith organ dysfunction could affect the occurrence of long-term RD merits further investigation.

In summary, our study emphasizes the importance of RD quantified evaluation. Nearly half of patients with BPPV had RD 1 week after successful CRP, and a third of them had moderate-to-severe RD. We propose that assessment of DHI and cVEMP at the initial diagnosis may provide prognostic value in predicting the likelihood of RD occurrence. DHI score >30 and bilateral cVEMP absence could increase the risk of short-term moderate-to-severe RD, which deserves further validation in other centers in clinical practice.

## Data Availability Statement

The raw data supporting the conclusions of this article will be made available by the authors, without undue reservation.

## Ethics Statement

The study was approved by the Ethics Committee of Shanghai Ninth People's Hospital, Shanghai Jiao Tong University School of Medicine (ethical approval number: 2018-128-T106). All enrolled patients signed the informed consent forms.

## Author Contributions

C-YJ contributed to the collection and assembly of data, data analysis, and interpretation. JW contributed to the provision of study materials, collection and assembly of data, data analysis, and interpretation. LS, X-HS, and HP contributed to the provision of study materials. QX contributed to the collection and assembly of data. S-CW contributed to data analysis and interpretation. J-RL contributed to administrative support, data analysis, and interpretation. YL contributed to the conception, design, data analysis, and interpretation. WC contributed to the conception, design, provision of study materials, data analysis, and interpretation. All the authors contributed to the manuscript writing and final approval of the manuscript.

## Funding

WC received grants from the National Natural Science Foundation of China (81401039), Shanghai Pujiang Program (18PJD023), Shanghai medical guidance program (17411964000), and the Clinical Research Program of Ninth People's Hospital affiliated to the Shanghai Jiao Tong University School of Medicine (JYLJ202003). C-YJ received grants for a scientific research project from Huangpu District, Shanghai, China (HLM202012).

## Conflict of Interest

The authors declare that the research was conducted in the absence of any commercial or financial relationships that could be construed as a potential conflict of interest.

## Publisher's Note

All claims expressed in this article are solely those of the authors and do not necessarily represent those of their affiliated organizations, or those of the publisher, the editors and the reviewers. Any product that may be evaluated in this article, or claim that may be made by its manufacturer, is not guaranteed or endorsed by the publisher.
