# Supplementary material for: Clinical and cVEMP Evaluation Predict Short-Term Residual Dizziness After Successful Repositioning in Benign Paroxysmal Positional Vertigo
Source: Front Med (Lausanne). 2022 May 24;9:881307. doi: 10.3389/fmed.2022.881307 (PMC9170995; doi:10.3389/fmed.2022.881307)
Supplement: Supplementary file 1 [file Data_Sheet_1.docx]

DHI and cVEMP evaluation predict short-term residual dizziness after successful repositioning in BPPV

Chun-Yan Jiang^†1,4^, Jing Wu^†1^, Liang Shu^1^, Xu-Hong Sun^1^, Hui Pan^1^, Qian Xu^1^, Si-Cheng Wu^3^, Jian-Ren Liu^*1^, Yun Li^*2^, Wei Chen^*1^

**Supplementary Table 1.**

**The** **detailed parameters of cVEMP in BPPV patients with bilateral elicited response**

|  | **Bilaterally present**  **cVEMP (*n*=129)** | **Group A**  **(*n*=73)** | **Group B**  **(*n*=41)** | **Group C**  **(*n*=15)** | ***p* value** |
| --- | --- | --- | --- | --- | --- |
| **cVEMP on the ipsilateral side** | | | | | |
| Threshold (dBnHL) | 100.6±7.4 | 101.1±6.9 | 99.9±8.0 | 100.0±8.0 | 0.817 |
| p1 Latency (ms) | 14.0±1.3 | 13.9±1.2 | 14.1±1.5 | 13.9±1.4 | 0.761 |
| n1 Latency (ms) | 22.3±1.8 | 22.3±1.8 | 22.4±1.9 | 22.5±1.4 | 0.486 |
| p1-n1 Amplitude (uV) | 83.8±44.8 | 85.0±44.7 | 81.8±47.0 | 83.2±41.8 | 0.895 |
| **cVEMP on the contralateral side** | | | | | |
| Threshold (dBnHL) | 100.2±7.4 | 100.6±7.5 | 100.1±7.3 | 98.4±7.2 | 0.546 |
| p1 Latency (ms) | 14.0±1.3 | 14.0±1.3 | 14.1±1.4 | 14.0±1.1 | 0.931 |
| n1 Latency (ms) | 22.3±1.7 | 22.3±1.5 | 22.3±1.4 | 22.6±2.8 | 0.912 |
| p1-n1 Amplitude (uV) | 83.0±51.3 | 85.2±57.3 | 77.2±44.2 | 88.7±40.1 | 0.548 |
| **IAD ratio, *n* (%)** |  |  |  |  | 0.503 |
| ≥25% | 19(14.7) | 11(15.1) | 7(17.1) | 1(6.7) |  |
| ≤-25% | 22(17.1) | 15(20.5) | 6(14.6) | 1(6.7) |  |
| -25%～25% | 88(68.2) | 47(64.4) | 28(68.3) | 13(86.6) |  |

Notes: cVEMP, cervical vestibular evoked myogenic potential; IAD, interaural amplitude difference.

**Supplementary Table 2.**

**The detailed parameters of oVEMP in BPPV patients with bilateral elicited response**

|  | **Bilaterally present oVEMP(*n*=87)** | **Group A**  **(*n*=51)** | **Group B**  **(*n*=24)** | **Group C**  **(*n*=12)** | ***p* value** |
| --- | --- | --- | --- | --- | --- |
| **oVEMP on ipsilateral side** | | | | | |
| Threshold (dBnHL) | 102.2±7.2 | 101.9±7.0 | 101.9±7.8 | 104.2±7.3 | 0.539 |
| n1 Latency (ms) | 11.1±1.0 | 11.1±1.0 | 10.7±0.9 | 11.4±1.3 | 0.213 |
| p1 Latency (ms) | 16.2±1.2 | 16.3±1.2 | 15.9±1.2 | 16.5±1.2 | 0.333 |
| n1-p1 Amplitude (uV) | 8.8±6.2 | 8.5±6.6 | 9.9±6.6 | 8.0±3.3 | 0.623 |
| **oVEMP on contralateral side** | | | | | |
| Threshold (dBnHL) | 101.6±7.6 | 102.3±7.0 | 99.8±7.5 | 102.1±9.9 | 0.310 |
| n1 Latency (ms) | 11.1±1.1 | 11.1±1.1 | 10.8±0.9 | 11.5±1.0 | 0.162 |
| p1 Latency (ms) | 16.3±1.2 | 16.4±1.1 | 15.9±1.4 | 16.7±1.3 | 0.053 |
| n1-p1 Amplitude (uV) | 8.4±5.0 | 7.6±4.6 | 10.3±5.9 | 8.3±4.0 | 0.176 |
| **IAD ratio, *n* (%)** |  |  |  |  | 0.255 |
| ≥25% | 12(13.8) | 6(11.8) | 2(8.3) | 4(33.3) |  |
| ≤-25% | 12(13.8) | 6(11.8) | 4(16.7) | 2(16.7) |  |
| -25%～25% | 63(72.4) | 39(76.5) | 18(75.0) | 6(50.0) |  |

Notes: oVEMP, ocular vestibular evoked myogenic potential; IAD, interaural amplitude difference.

**Supplementary Table 3.**

**Logistic regression analyses for RD and its severity in patients with BPPV when cVEMP was dichotomized into two subgroups**

| **Variables** | **β** | **OR** | **95%CI** | ***p* value** |
| --- | --- | --- | --- | --- |
| **Model 1: dependent factor: Group (B+C) vs. Group A** | | | | |
| DHI score>30 | 0.741 | 2.099 | 1.217~3.619 | **0.008^**^** |
| cVEMP absence on either side | 0.565 | 1.760 | 1.013~3.059 | **0.045^*^** |
| **Model 2: dependent factor: Group B vs. Group A** | | | | |
| None |  |  |  |  |
| **Model 3: dependent factor: Group C vs. Group A** | | | | |
| DHI score> 30 | 1.558 | 4.747 | 1.985~11.356 | **<0.001^***^** |
| cVEMP  Bilateral presence  Absence on either side | 1.143 | 1.0 (reference)  3.136 | 1.390~7.073 | **0.006^**^** |

Notes: RD, residual dizziness; BPPV, benign paroxysmal positional vertigo; DHI, dizziness handicap inventory; cVEMP, cervical vestibular evoked myogenic potential; β represents regression coefficient; OR, odds ratio; CI, confidential interval; * *p* < 0.05; ** *p* < 0.01; *** *p* < 0.001.
